# Cardiorenal syndrome in incident peritoneal dialysis patients: What is its effect on patients’ outcomes?

**DOI:** 10.1371/journal.pone.0218082

**Published:** 2019-06-07

**Authors:** Yanmei Xue, Baozhen Xu, Chunyan Su, Qingfeng Han, Tao Wang, Wen Tang

**Affiliations:** 1 Department of Nephrology, Peking University Third Hospital, Beijing, China; 2 School of Chinese Integrative Medicine, Hebei Medical University, Shijiazhuang, China; 3 Department of Nephrology, The First Hospital of Hebei Medical University, Shijiazhuang, China; Universidade Estadual Paulista Julio de Mesquita Filho, BRAZIL

## Abstract

**Background:**

Peritoneal dialysis (PD) is increasingly used for long-term management of Cardiorenal Syndrome (CRS). We compared outcomes in incident PD patients according to their baseline heart failure status.

**Methods:**

This retrospective cohort study evaluated all-cause and cardiovascular mortality in incident PD patients with different heart failure status (non-CRS, acute heart failure [AHF], type II CRS, type IV CRS) who started PD between 2006 and 2016 in the Peking University Third Hospital.

**Results:**

Of 748 patients included in the study, there were 466 (62.3%), 214 (28.6%), 27 (3.6%), and 41 (5.5%) patients in the non-CRS, AHF, type II CRS and type IV CRS groups, respectively. Patients with CRS were older (p<0.001), with more diabetes mellitus (p<0.001), coronary heart history (p<0.001), higher estimated glomerular filtration rate (eGFR) (p<0.001), lower serum creatinine (p<0.001) and phosphorus levels (p = 0.003) compared to non-CRS patients. Respective all-cause survival rates for patients with non-CRS, AHF, type II CRS and type IV CRS were 90.6%, 87.1%, 85.2% and 84.8% at 1 year, and 63.1%, 47.7%, 27.3% and 35.1% at 5 years (p<0.001). The corresponding figures for cardiovascular survival were 93%, 92%, 84% and 81% at 1 year, and 67%, 59%, 55% and 54% at 5 years (p<0.001). However, after adjusting for confounding factors, the presence of CRS was not independently associated with all-cause mortality whereas type IV CRS (HR 2.10, 95% CI 1.03–4.28, p = 0.04) was associated with higher cardiovascular mortality as compared to without CRS.

**Conclusion:**

Incident PD patients with different types of CRS had higher rates of both all-cause and cardiovascular mortality compared with patients without CRS. However, these observed adverse outcomes may be related to associated older age and higher prevalence of comorbidities, rather than CRS per se, except for type IV CRS, treatment strategies to reduce high cardiovascular CVD mortality may needed.

## Introduction

Heart failure (HF) is a severe global public health problem characterized by an increasing prevalence in older populations, high mortality, and an appreciable financial burden on the healthcare system. The reported estimated prevalence of heart failure in China is 0.9% [1]. Despite improvements in therapies in recent decades, the mortality rate in patients with HF has remained high [2].

The term cardiorenal syndrome (CRS) was defined as a pathophysiological disorder of the heart and kidneys in which acute or chronic dysfunction in one organ may induce acute or chronic dysfunction in the other organ [3–5]. The CRS was classified five types: type I, acute cardiorenal syndrome; type II, chronic cardiorenal syndrome; type III, acute renocardiac syndrome; type IV, chronic renocardiac syndrome; type V, secondary cardiorenal syndrome [6,7]. Chronic renal dysfunction is common in HF patients, either as a secondary consequence, as seen in type II CRS, or as a primary cause, as seen in type IV CRS [8]. Although ultrafiltration modalities like hemofiltration have been successfully applied in decompensated heart failure with a favorable efficacy, it is less suitable to use this therapy for chronic treatment of HF with CRS due to a requirement for complicated equipment, high costs, vascular access-related problems and the need for continuous anticoagulation[9]. Peritoneal dialysis (PD) may represent a better treatment option in maintenance therapy due to its continuous, slow and more physiologic ultrafiltration [10,11]. Another reason that patients preferred to PD was because it can be completed at home [12]. However, the outcomes of patients with CRS treated by PD have received limited evaluation.

Several small series and single center experiences have shown that PD therapy is associated with improved heart functional status, reduced hospitalization rate and possibly decreased mortality rate in patients with heart failure [13–15]. However, these studies were limited by small sample sizes, short follow-up durations, a lack of appropriate control groups, inclusion of prevalent PD patients, inadequate adjustment for confounding and failure to adequately characterize CRS type. Therefore, in the present study, we compared unadjusted and adjusted all-cause and cardiovascular survival outcomes in a large cohort of incident PD patients according to baseline heart failure status (non-CRS, AHF, type II CRS and type IV CRS).

## Materials and methods

In this retrospective cohort study, all incident PD patients who started PD therapy from January 1, 2006 to December 31, 2016 in Peking University Third Hospital were included. During and after data collection, the authors could not identify individual participants as the patients’ names were replaced by PDID during the data collection and analyses. This study was approved by the Medical Scientific Research Ethical Committee of Peking University Third Hospital. The exclusion criteria were as follows: (1) patients who could not be assigned to any CRS group according to their clinical history; (2) patients with active malignancy; (3) patients who were lack of medical history about cardiac function. Patients were followed until death, cessation of PD or end of study as of February 28, 2018. All procedures performed in studies involving human participants were in accordance with the ethical standards of the institutional and/or national research committee and with the 1964 Helsinki declaration and its later amendments or comparable ethical standards.

In our center, patients need to be hospitalized to department of nephrology for performing PD catheter implantation. Therefore, patients’ detailed kidney disease history as well as other disease history especially heart disease history were recorded when admitted. During the study period, patients’ hospitalization medical charts were carefully reviewed to obtain the data for kidney disease as well as diagnosis of heart failure when admitting. The evidence for categorization of heart failure status were also obtained from patients’ hospitalization charts. In the present study, during the classification process, two assessors (two nephrologists) used the same chart to perform all assessments for study participants separately and independently. And the final results were reviewed by a senior nephrologist if there were discrepancy exist. The final type decision were discussed by the three nephrologists together. Accordingly, patients were divided into four groups: 1) non-CRS group, defined as no symptoms or evidence of heart failure at the initiation of PD; 2) acute heart failure (AHF) group, defined as with kidney failure and acute onset of symptoms of heart failure (like with dyspnea, short of breath and pulmonary edema at the initiation of PD) but without any history of chronic heart failure or acute heart failure episode; 3) type II CRS (chronic cardiorenal syndrome), whereby patients had a chronic heart failure history (CHF) and chronic renal failure history (history of chronic renal failure is shorter than CHF). Additionally, according to Ronco’s definition [4], based on description of medical chart, only the progress of renal failure is considered caused by chronic abnormalities in cardiac function were defined as type II CRS, specifically, in the present study, all the typeIICRS patients were those who were with refractory heart failure and were referred to nephrologists by cardiologists to seek for treatment of heart failure by peritoneal dialysis. and 4) type IV CRS, whereby patients had a chronic kidney disease history and chronic heart failure (history of chronic kidney disease is longer than CHF history). Additionally, according to Ronco’s definition [4], based on description of medical chart, only the condition of primary CKD contributing to decreased cardiac function were considered as type IV CRS. Patients’ demographics characteristics, primary disease, comorbidities, and other baseline data at the initiation of PD (which is the last pre-dialysis measurement prior to dialysis) were also obtained. In particular, pre-dialysis serum creatinine prior to peritoneal dialysis were used to calculate estimated glomerular filtration rate (eGFR). eGFR was estimated by the Chronic Kidney Disease Epidemiology Collaboration (CKD-EPI) creatinine equation[16]. All patients at this center were dialyzed with glucose-based dialysis solutions (Dianeal, Baxter, Guangzhou, China) and were received DAPD (daytime ambulatory peritoneal dialysis) or CAPD (continuous ambulatory peritoneal dialysis) therapy except a few of patients were received interim APD (automated peritoneal dialysis) treatment during hospitalization.

The primary outcomes were all-cause patient survival (censored for renal function recovery, loss to follow-up, renal transplantation and end of study) and cardiovascular survival (censored for non-cardiovascular death, renal function recovery, loss to follow-up, renal transplantation and end of study). Cardiovascular death was defined as death from cardiovascular disease, defined as acute myocardial infarction, sudden death, heart failure, aortic aneurysm rupture, cerebrovascular accident, and other cardiovascular reasons.

## Statistical analysis

Continuous variables were presented as the mean±standard deviation (SD) or median (interquartile range, IQR). Qualitative data were expressed as absolute numbers and percentages. One way ANOVA or Chi-square tests were used in comparison for patients in different groups as appropriate. Mortality and cardiovascular mortality were assessed by Kaplan–Meier and multivariate Cox proportional hazards model analyses in which all the significant variables (p < 0.1) from the univariate Cox analysis were included. Statistical analysis was performed using SPSS software, version 22.0 (SPSS Inc., Chicago, IL, USA). P values less than 0.05 were considered statistically significant.

## Results

### Patient characteristics

A total of 748 patients were included in this study. The median follow-up time was 6.23 (IQR 2.423–11.652) years. There were 466 (62.3%), 214 (28.6%), 27 (3.6%) and 41 (5.5%) patients in non-CRS group, AHF group, type II CRS and type IV CRS groups, respectively. Comparison of baseline characteristics among the four groups were shown in Table 1.

**Table 1:** Comparison of clinical characteristics among different CRS groups.

Variable	Group				P
	non-CRS (n = 466)	AHF (n = 214)	II CRS (n = 27)	IV CRS (n = 41)	
Age (years)	57.4±16.5	60.3±16.1	65.7±13.8^a^	67.9±12.6^a^^,^^b^	<0.001
Gender (male, %)	239(51%)	105(49%)	10(37%)	21(51%)	0.53
Height (cm)	163.7±8.3	164.1±8.4	160.6±7.5	163.2±8.0	0.78
BMI (kg/ m^2^)	23.5±4.0	24.0±3.7	24.5±4.7	23.6±3.7	0.40
Creatinine (μmol/L)	853±347	784±349	587±283^a^^,^^b^	562±270^a^^,^^b^	<0.001
Urea (mmol/L)	30.9±10.4	29.6±10.2	27.6±11.2	26.7±11.3	0.03
Albumin (g/L)	36.8±5.3	33.3±4.6	35.3±4.6	35.7±5.0	<0.001
Hemoglobin (g/L)	86.7±18.7	78.9±17.9	85.7±14.6	87.8±14.6^b^	<0.001
Calcium (mmol/L)	1.9±0.3	1.8±0.3	2.0±0.2^b^	1.9±0.2	<0.001
Phosphorous (mmol/L)	2.0±0.5	1.9±0.5	1.7±0.4^a^	1.7±0.5^a^	0.003
eGFR[ml/(min∙1.73m ^2^)]	5.5±2.4	6.2±3.5	8.4±4.4^b^	9.1±4.5^a^^,^^b^	<0.001
SBP(mmHg)	148.3±21.7	152.2±24.6	151.0±25.7	144.1±24.9	0.09
DBP(mmHg)	84.4±13.9	84.0±15.1	77.5±14.5	81.2±14.1	0.06
Diabetes mellitus	146(31%)	103(48%)^a^	15(56%)	21(51%)	<0.001
CHD history	60(13%)	50(23%)^a^	9(33.3%)^a^	18(44%)^a^^,^^b^	<0.001
Cerebral hemorrhage	14(3%)	4(2%)	0(0%)	2(5%)	0.53
Primary disease					<0.001
DN	124(27%)	90(42%)	14(52%)	17(42%)	
HBP	64(14%)	25(12%)	7(25.9%)	10(24%)	
PKD	20(4%)	4(2%)	0(0%)	1(2%)	
CGN	177(38%)	62(29%)	4(15%)	4(10%)	
CIN	54(12%)	12(6%)	2(7%)	2(5%)	
Unknown	17(4%)	13(6.1%)	0(0%)	4(10%)	
Others	10(2%)	8(4%)	0(0%)	3(7%)	

CRS: cardiorenal syndrome; AHF: acute heart failure; eGFR: estimated glomerular filtration rate; SBP: systolic blood pressure; DBP: diastolic blood pressure; CHD: coronary heart disease; DN: diabetic nephropathy; HBP: hypertension; PKD: polycystic kidney disease; CGN: chronic glomerulonephritis; CIN: chronic interstitial nephritis; BMI: Body Mass Index

II CRS: type II CRS, patients who had a history of chronic congestive heart failure and progressive renal failure

IV CRS: type IV CRS, patients who had a history of chronic renal dysfunction causing chronic heart failure

Note:

^a^: compared to non CRS group, p<0.05;

^b^: compared to AHF group, p<0.05

In general, the patients with CRS were older (p<0.001), with more diabetes mellitus (p<0.001) and coronary heart history (p<0.001) as compared to the non-CRS group. Patients with CRS had higher eGFR (p<0.001), but lower serum creatinine (p<0.001), urea (p = 0.03) and phosphorus levels (p = 0.003) compared to the non-CRS group. Patients’ serum calcium and hemoglobin levels were significantly lower in the AHF patients as compared to patients with type II CRS and type IV CRS (p<0.001). There were no significant differences in gender distribution, cerebral vascular disease history, or systolic and diastolic blood pressures among the four groups (p>0.05). The etiology of type II CRS were ischemic heart disease (36%), Rheumatic heart disease (12%), hypertensive heart disease (32%), diabetic cardiomyopathy (12%) and others (8%). There were no significant difference in left ventricular ejection fraction between type II CRS and type IV CRS (54.3±14.8% vs.53.8±14.8%, p = 0.93).

### Comparison of All-cause mortality among different CRS groups

**Fig 1:**
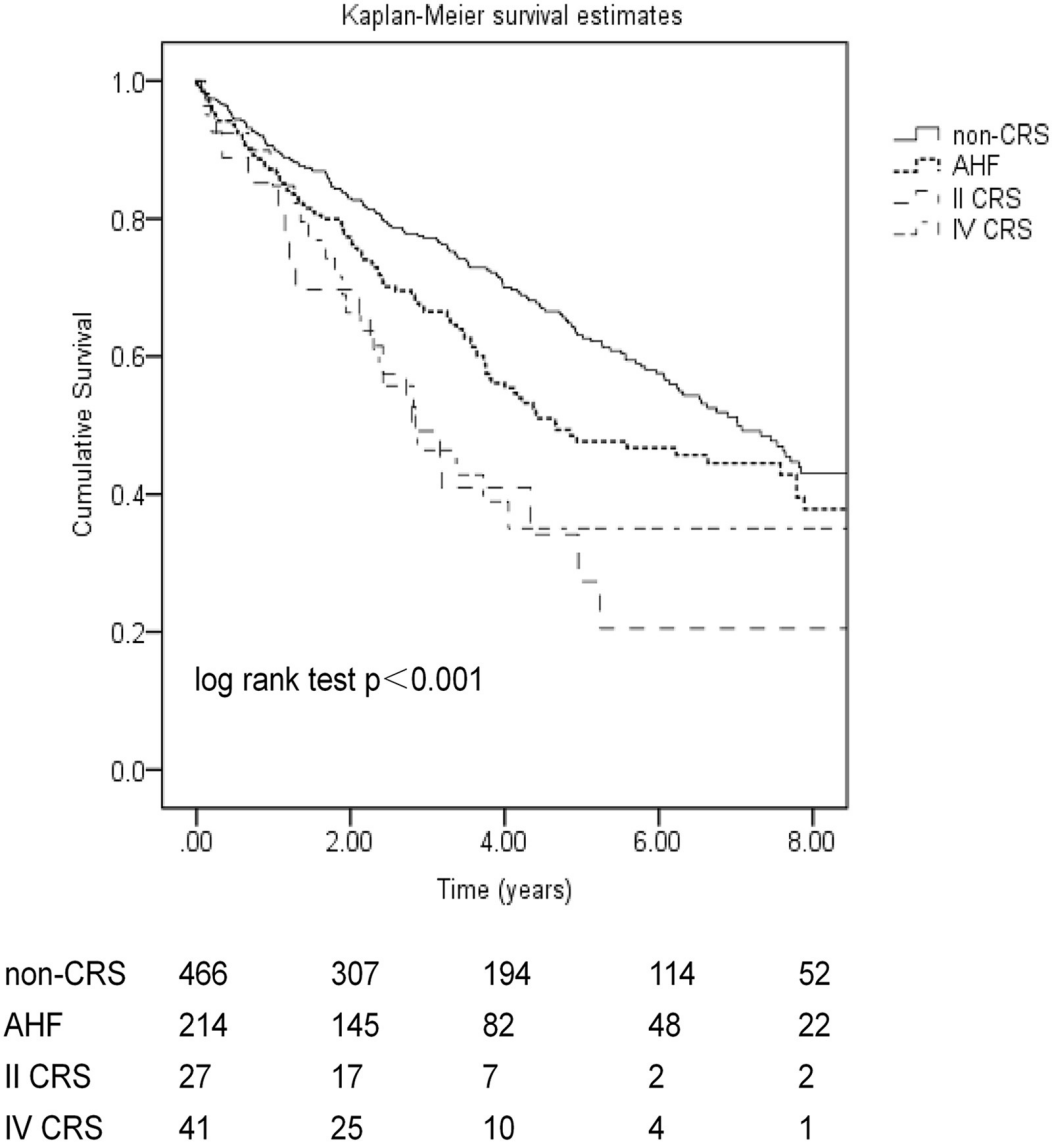
Kaplan-Meier survival curves in incident peritoneal dialysis patients with different cardiorenal syndrome. CRS: cardiovascular renal syndrome; AHF: acute heart failure; II CRS: type II CRS, patients who had a history of chronic congestive heart failure and progressive renal failure; IV CRS: type IV CRS, patients who had a history of chronic renal dysfunction causing chronic heart failure.

Death occurred in 317 (42.4%) patients. A total of 93 (12.4%) patients transferred to hemodialysis. Forty-one patients (5.5%) underwent renal transplantation and five (0.7%) patients had recovery of renal function during follow-up. Loss to follow-up occurred in 90 patients (12%) and 24 (3.2%) patients transferred to other dialysis centers. The causes of death for non-CRS and other forms of CRS (AHF, type II CRS, type IV CRS) patients were cardiovascular disease (34.7% vs 44.1%, 44.4%and 50.5%), peritonitis (12% vs 4.9%, 11.1% and 0%), multiple organ failure (9.8% vs 11.8%, 11.1% and 0%), infection (14.5% vs 12.7%, 11.1% and 20.8%), tumor (7.5% vs 4.9%, 5.6% and 0%), withdrawal (2.9% vs 4.9%, 0% and 4.2%), gastrointestinal bleeding (7.5% vs 3.9%, 0% and 4.2%), respiratory failure (8.1% vs 3.9%, 5.6% and 8.3%) and unknown reason (8.1% vs 8.8%, 11.1% and 12.5%),respectively. There were no statistically significant differences in the causes of death among the groups (p = 0.82). The mortality rate in the non-CRS group was significantly lower compared with the CRS groups (log-rank test, p<0.001) (Fig 1). Respective survival rates in the non-CRS, AHF, type II CRS and type IV CRS groups were 90.6%, 87.1%, 85.2% and 84.8% at 1 year; 77.2%, 76.8%, 69.1% and 66.3% at 2 years; 77.2%, 66.5%,49.2% and 46.4% at 3 years; and 63.1%, 47.7%, 27.3% and 35.1% at 5 years (p<0.001). The median survival of patients with non-CRS (7.03 years, 95% CI 6.16–7.89 years) was better as compared to that of AHF patients on dialysis (4.66 years, 95% CI 2.80–6.53 years, p = 0.02), patients with type II CRS (2.80 years, 95% CI 1.89–3.70 years, p<0.001) and patients with type IV CRS (2.85 years, 95% CI 1.65–4.05 years, p<0.001), respectively. Univariate Cox regression analysis showed that AHF patients (HR 1.33, 95% CI 1.04–1.70, p = 0.02), type II CRS patients (HR 2.27, 95% CI 1.40–3.70, p = 0.001) and type IV CRS patients (HR 2.10, 95% CI 1.37–3.23, p = 0.001) had significantly higher mortality risks compared with non-CRS patients. However, using multivariable Cox proportional hazards model analysis, CRS (AHF, type II CRS, type IV CRS) was not significantly and independently associated with patient survival on peritoneal dialysis compared with non-CRS patients after adjusting for other confounding factors, including age (p<0.001), diabetes mellitus (p = 0.07), serum albumin (p = 0.003), serum calcium (p<0.001) and eGFR (p = 0.30) (Table 2).

**Table 2:** Cox proportional hazards model for all-cause mortality rate in incident peritoneal dialysis patients with different cardiorenal syndrome.

Variable	Univariate Cox regression		Multivariate Cox regression	
	HR [95%CI]	p	HR [95%CI]	P
CRS group*		<0.001		0.285
Non-CRS	reference			
AHF	1.33[1.04–1.70]	0.02	1.19[0.91–1.56]	0.21
II CRS	2.27[1.40–3.70]	0.001	1.47[0.87–2.48]	0.15
IV CRS	2.10[1.37–3.23]	0.001	1.39[0.86–2.23]	0.18
Age (per 1-year increase)	1.05[1.04–1.06]	<0.001	1.05 [1.04–1.06]	<0.001
Gender (female)	1.09[0.88–1.36]	0.44		
Creatinine (per 1-umol/l increase)	0.99[0.98–0.99]	<0.001	1.00[1.00–1.00]	0.66
Urea (per 1-mmol/l increase)	0.98 [0.97–0.99]	<0.001	1.01[1.00–1.03]	0.19
Albumin (per 1-g/l increase)	0.95[0.93–0.97]	<0.001	0.94[0.92–0.96]	<0.001
Hemoglobin (per 1-g/l increase)	1.00[1.00–1.01]	0.02	1.00[0.99–1.01]	0.83
Calcium (per 1-mmol/l increase)	2.12[1.51–2.99]	<0.001	2.65[1.79–3.93]	<0.001
Phosphorus (per 1-mmol/l increase)	0.59[0.48–0.73]	<0.001	1.07[0.82–1.40]	0.60
eGFR (per 1-ml/min.1.73 m ^2^ increase)	1.09[1.06–1.12]	<0.001	1.043[0.97–1.10]	0.30
SBP (per 1-mmHg increase)	0.99[0.99–1.00]	0.65		
DBP (per 1-mmHg increase)	0.98[0.97–0.98]	<0.001	1.00[0.99–1.01]	0.62
Diabetes(yes)	1.83 [1.47–2.29]	<0.001	1.26[0.99–1.61]	0.07
CHD history(yes)	1.53 [1.27–1.84]	<0.001	0.97[0.74–1.27]	0.83

Multivariate Cox’s proportional hazards model included all the significant variables (p < 0.1) from the univariate analysis. CI: confidence interval; AHF: acute heart failure; CRS: cardiorenal syndrome; CHD: coronary heart disease; eGFR: estimated glomerular filtration rate; SBP: systolic blood pressure; DBP: diastolic blood pressure

II CRS: type II CRS, Patients who had a history of chronic congestive heart failure and progressive renal failure

IV CRS: type IV CRS, patients who had a history of chronic renal dysfunction causing chronic heart failure

* compared to non-CRS

### Comparison of cardiovascular (CVD) mortality among different CRS groups

**Fig 2:**
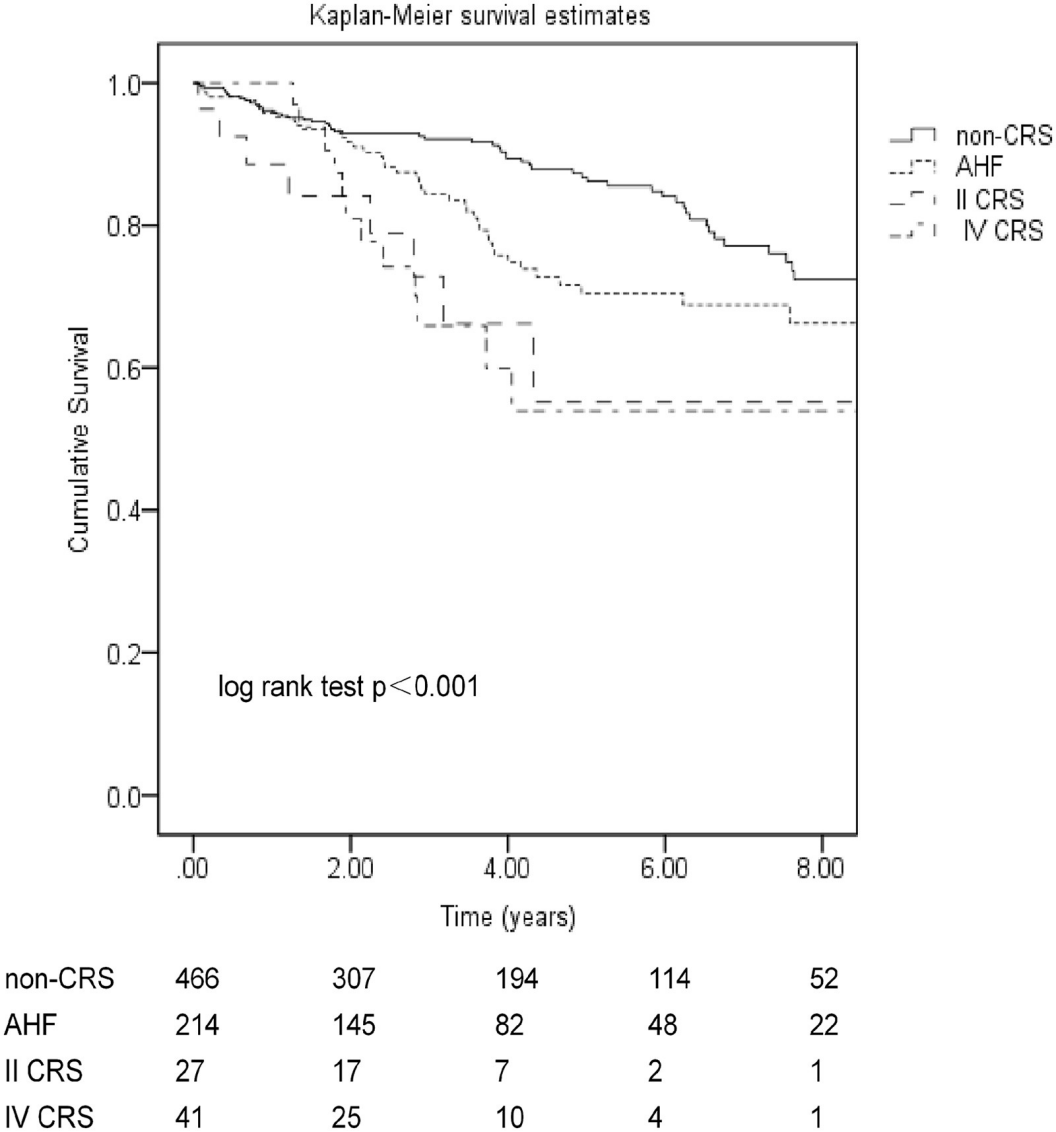
Kaplan-Meier cardiovascular death-free survival curves in incident peritoneal dialysis patients with different cardiorenal syndrome. CRS: cardiovascular renal syndrome; AHF: acute heart failure; II CRS: type II CRS, patients who had a history of chronic congestive heart failure and progressive renal failure; IV CRS: type IV CRS, patients who had a history of chronic renal dysfunction causing chronic heart failure.

Cardiovascular disease was the dominant cause of death, occurring in 125 patients (39.4%). The causes of cardiovascular death for non-CRS and other forms of CRS (AHF, type II CRS, type IV CRS) patients were acute myocardial infarction (5.8% vs 4.9%, 5.6% and 8.3%), sudden death (9.8% vs 17.6%, 11.1% and 29.2%), heart failure (5.2% vs 2.9%, 0% and 12.5%),dissecting (0% vs 1.0%, 0% and 0%), cerebral hemorrhage(2.9% vs 5.9%, 5.6% and 0%), cerebral infarction (4.6% vs 7.8%, 11.1% and 0%), other cardiac reasons (1.2% vs 0%, 0% and 0%), respectively. The cardiovascular mortality rate in the non-CRS group was significantly lower compared to the CRS group (log-rank test, p<0.001) (Fig 2). Respective cardiovascular survival rates in the four groups were 93%, 92%, 84% and 81% at 1 year; 92%, 85%, 73% and 66% at 2 years; 90%, 76%, 66% and 61% at 3 years; and 67%, 59%, 55% and 54% at 5 years. The mean cardiovascular survival time of patients with non-CRS (9.75 years, 95% CI 9.24–10.26 years) was better than that of AHF patients on dialysis (8.64 years, 95% CI 7.88–9.40 years, p = 0.007), patients with type II CRS on dialysis (7.10 years, 95% CI 4.97–9.24 years, p = 0.002) and patients with type IV CRS on dialysis (5.77years, 95% CI 4.57–6.97 years, p<0.001). Univariate Cox regression analysis showed that AHF patients (HR 1.68, 95% CI 1.14–2.48, p = 0.008), type II CRS patients (HR 2.97, 95% CI 1.42–6.22, p = 0.004) and type IV CRS patients (HR 3.11, 95% CI 1.67–5.80, p<0.001) had significantly higher cardiovascular mortality risk as compared with non-CRS patients. Using multivariable Cox proportional hazards model analysis, in general, CRS types was not independently associated with cardiovascular mortality on peritoneal dialysis after adjusting for other confounding factors, including age (p = 0.001), diabetes mellitus (p = 0.05), coronary heart disease (p = 0.01), serum albumin (p = 0.001), serum calcium (p = 0.01). (Table 3). However, as compared to non-CRS group, patients with type IV CRS was significantly associated with higher cardiovascular mortality (HR 2.10, 95% CI 1.03–4.28, p = 0.04) whereas both AHF (HR 1.37, 95% CI 0.90–2.08, p = 0.15) and type II CRS (HR 1.82, 95% CI 0.91–4.14, p = 0.15) were not significantly associated with cardiovascular mortality after adjusting for other confounding factors.

**Table 3:** Cox proportional hazards model for cardiovascular-cause mortality in incident peritoneal dialysis patients with different cardiorenal syndrome.

Variable	Univariate Cox regression		Multivariate Cox regression	
	HR [95%CI]	p	HR [95%CI]	P
CRS group*		<0.001		
Non-CRS	Reference			
AHF	1.68[1.14–2.48]	0.008	1.37[0.90–2.08]	0.15
II CRS	2.97[1.42–6.22]	0.004	1.82[0.81–4.14]	0.15
IV CRS	3.11[1.67–5.80]	<0.001	2.10[1.03–4.28]	0.04
Age (per 1-year increase)	1.04[1.03–1.06]	<0.001	1.034[1.01–1.05]	0.001
gender (male)	0.94[0.66–1.34]	0.74		
Creatinine (per 1-umol/l increase)	0.99[0.99–1.00]	<0.001	1.00[1.00–1.00]	0.85
Urea (per 1-mmol/l increase)	0.98[0.96–0.99]	0.01	1.00[0.98–1.03]	0.95
Albumin (per 1-g/l increase)	0.94[0.91–0.97]	<0.001	0.94[0.90–0.97]	0.001
Hemoglobin (per 1-g/l increase)	1.01[1.00–1.02]	0.12		
Calcium (per 1-mmol/l increase)	1.84[1.08–3.15]	0.03	2.34[1.25–4.38]	0.01
Phosphorus (per 1-mmol/l increase)	0.69[0.49–0.95]	0.02	1.39[0.91–2.11]	0.13
eGFR (per 1-ml/min.1.73 m ^2^ increase)	1.10[1.05–1.15]	0.001	1.02[0.92–1.12]	0.72
SBP (per 1-mmHg increase)	0.99[0.99–1.00]	0.11		
DBP (per 1-mmHg increase)	0.97[0.96–0.99]	<0.001	0.99[0.97–1.00]	0.09
Diabetes(yes)	2.34[1.64–3.33]	<0.001	1.48 [1.00–2.20]	0.05
CHD history(yes)	1.87[1.51–2.32]	<0.001	1.49[1.10–2.02]	0.01

Multivariate Cox’s proportional hazard model included all the significant variables (p < 0.1) from the univariate analysis.CI: confidence interval; AHF: acute heart failure; CRS: cardiorenal syndrome; CHD: coronary heart disease; eGFR: estimated glomerular filtration rate; DBP: diastolic blood pressure

II CRS: type II CRS, patients who had a history of chronic congestive heart failure and progressive renal failure

IV CRS: type IV CRS, patients who had a history of chronic renal dysfunction causing chronic heart failure

*compared to non-CRS

## Discussion

Our results demonstrated that incident PD patients with acute heart failure, type II CRS and type IV CRS had higher all-cause and cardiovascular mortality compared with patients without CRS at a single PD center in China. However, patients with CRS were older and had more comorbidities. After adjusting for these confounding factors at baseline, with different types of CRS were not significantly associated with all-cause mortality. However, patients with type IV CRS were significantly associated with higher cardiovascular mortality on peritoneal dialysis compared with patients without CRS.

In our study, the one-year all-cause survival rate in type II CRS was 85.2%, which appeared to be significantly higher than that so far reported for patients with HF treated with conservation treatment[17]. Various studies have reported a significant improvement in one-year survival rate in HF following PD therapy [13,14,18,19]. The median all-cause survival time in type II CRS patients was 2.80 years (one-year survival rate of 85.2%), similar to several other reports of one-year survival rates of 85% in Courivaud et al.[13] 82% in Bertoli et al.[14], but apparently higher than the reported 58% in Kunin et al.[18] and Sanchez et al.[19]. The variable survival rates may have been due to differences in HF severity, HF etiologies and residual renal function. It should be noted the survival difference could also be generated by the different dialysis dose since most patients with CRS type II of up mentioned studies had relatively high GFR therefore treated by only peritoneal ultrafiltration (PUF) (a single exchange per day could be possible) but patients in the present study had relatively lower values of GFR thus a relatively higher dose of PD exchange would be given to these patients to maintain dialysis adequacy although we do not have the detailed data.

Unlike previous studies that considered HF as a whole or limited their evaluation to only type II CRS without specifically investigating other types of CRS, the present study included patients with both type IV and type II CRS in an incident PD cohort thereby allowing comparison of outcomes between these two different CRS types.

Patients with CRS had higher baseline eGFR values at PD commencement than those without CRS. This observation most likely reflects the fact that patients with CRS experienced indications for dialysis commencement (such as refractory fluid overload or heart failure) at an earlier stage of their chronic kidney disease (CKD) journey than patients without CRS. Although we adjusted for baseline eGFR in the multivariable analyses, the possibility of lead time bias cannot be excluded. Similar to previous studies [20–23], higher eGFR at dialysis commencement was associated with higher mortality. This may be related to the fact that patients who started dialysis earlier at higher levels of eGFR were more likely to have different reasons for commencing dialysis (such as associated illnesses, poor volume control, etc.). Indeed, patients with CRS were more likely to be older and have diabetes, coronary artery disease and diabetic kidney disease than patients without CRS. Although we adjusted for baseline eGFR, age and comorbidities, the possibility of residual confounding cannot entirely be excluded.

The strengths of this study include its large sample size, long-term follow-up, more precise subcategorization of CRS and use of multivariable analyses to adjust for potentially confounding factors. Weighed against these strengths, the study had several limitations. First, patients included in this study were recruited from a single tertiary academic hospital in China, thereby raising the possibility of ascertainment bias. Second, due to the retrospective design, some detailed management information could not be obtained, such as the use of a calculated GFR but not measured GFR which is important for PD, the absence of a quantification of diuresis and lack of detailed PD treatment regimen. Third, although we carefully attempted to categorize CRS type, we could not rule out correlation but not causation of kidney disease and cardiac disease. Thus the possibility of misclassification bias cannot be excluded especially on type IV CRS. In particular, the AHF group defined in this study is inconsistent with type I CRS defined by Ronco. Besides, in spite that we tried to exclude patients with history of chronic heart failure in AHF group, we still could not rule out the possibility that patients with underlining chronic heart failure and with a new onset of AHF were included. Fourth, patients with CRS commenced dialysis at higher eGFR levels than those who did not have CRS. Whilst lead time bias is possible, this would have likely reduced any apparent survival disadvantage of CRS. Fifth, the numbers of death events in the CRS sub-groups were relatively small, which would have reduced statistical power. Finally, although we attempted to adjust for a range of demographic, clinical and laboratory characteristics, residual confounding remains possible.

In conclusion, our study demonstrated that incident PD patients with CRS had higher rates of both all-cause and cardiovascular mortality compared with patients without CRS. These adverse outcomes of all-cause mortality were no longer apparent following adjustment for age and comorbidities, suggesting that these factors rather than CRS per se may account for the observed higher mortality rates in patients with CRS except for type IV CRS, more treatment strategies to reduce high CVD mortality in this groups of patients may needed.

## Supporting information

S1 File Data set for this study. (XLSX)
